# Biodiversity science and biosurveillance are fellow travelers

**DOI:** 10.1093/biosci/biaf091

**Published:** 2025-06-24

**Authors:** Timothée Poisot, Daniel J. Becker, Michael D. Catchen, Rory Gibb, Paloma H.F. Shimabukuro, Colin J. Carlson

**Affiliations:** Département de Sciences Biologiques and with the Québec Centre for Biodiversity Science at the Université de Montréal, in Montréal, Québec, Canada; School of Biological Sciences at the University of Oklahoma, in Norman, Oklahoma, in the United States; Département de Sciences Biologiques and with the Québec Centre for Biodiversity Science at the Université de Montréal, in Montréal, Québec, Canada; Department of Genetics, Evolution, and Environment, at University College London, in London, England, in the United Kingdom; Fundação Oswaldo Cruz, in Rio de Janeiro, Brazil, and with the Vector Data Support Group at the Global Biodiversity Information Facility, in Copenhagen, Denmark; Yale University School of Public Health, in New Haven, Connecticut, in the United States

The failure to meet the Aichi targets to alleviate global biodiversity decline (Nature 2020) was a wake-up call to the biodiversity monitoring community (Tittensor et al. 2014, Maney et al. 2024). In response, emphasis shifted toward translating research into action (Mace et al. 2018) and ensuring that the outcomes of these actions are spatially explicit and can be tracked through time. Leading up to the adoption of the Kunming-Montréal Global Biodiversity Framework (KM-GBF), the field reoriented around the systematic integration of monitoring actions (Leadley et al. 2022); this included, notably, mapping of the indirect and direct drivers of biodiversity change, quantifying their relative contributions to the indicators tracked as part of specific targets and goals at the scale of genes, species, and ecosystems. Although it is too early to know whether we will meet these new targets, it is clear that biodiversity monitoring is now federated around the need to guide and encourage concrete actions (Gonzalez and Londoño 2022). This change has resulted in a realignment, not only of the research directions but also of the integration between research, data ecosystems (Griffith et al. 2024, Royaux et al. 2024), and technologies for trends detection and forecasting (Gonzalez et al. 2023b).

The One Health approach sees advances in the health of ecosystems, humans, plants, and animals as mutually beneficial. In recent years, the One Health community has been calling for more integration of environmental expertise in what was traditionally a veterinary medicine–dominated field. The biodiversity monitoring community is now in a much better position to provide tools, data, and expertise toward assisting with, for example, the recent call by the One Health quadripartite secretariat to mainstream biodiversity and environmental expertise (The Quadripartite Organizations 2022). The rationale behind this mainstreaming is simple: By more effectively maintaining the health of ecosystems, downstream intervention on human and animal health may be easier and more effective. This mainstreaming would establish biodiversity conservation as one of the (many) tools toward successful interventions for health (understood in the present article in its holistic One Health definition: actions benefiting ecosystems, humans, animals, and plants), as well as provide data relevant to questions surrounding the risk of zoonotic pathogen spillover and pandemics as stemming from biodiversity-related processes at the human–wildlife interface (Frederick et al. 2021, Desvars-Larrive et al. 2024).

In the present article, we identify three lessons learned from the field of biodiversity monitoring that are relevant to biosurveillance (i.e., the monitoring and detection of pathogens in humans, plants, and animals). We frame these advances in terms of concrete steps that can be taken to further integrate the two fields. We then articulate a vision for how this work can be reused and extended to assist with biosurveillance, in a way that would strengthen the integration and effectiveness of both fields.

## Lesson learned: Essential biodiversity variables facilitate translation of research into policy work

The KM-GBF monitoring framework, adopted in 2022, includes specific lists of headline, global, component, and complementary indicators, as well as the opportunity for these indicators to be supplemented by national and subnational indicators. The establishment, measurement, and classification of these indicators benefits from previous work on essential biodiversity variables (EBVs; Pereira et al. 2013). Made to facilitate the aggregation, sharing, and use of biodiversity data, EBVs serve to calculate and report on biodiversity indicators. They are designed to support the monitoring and, for this reason, contribute to the monitoring framework of the KM-GBF (Schmeller et al. 2017). EBVs are also interoperable (Hardisty et al. 2019): Multiple EBVs can be combined or can share data in a way that allows economies of scale for monitoring. EBVs are also a flexible framework, with recent proposals to expand them to the monitoring of ecosystem services (Schwantes et al. 2024). Borne out of a collaborative design process, EBVs have been tailored to specific ecosystems, such as freshwater (Turak et al. 2017), or extended to novel data types such as species range and functional traits (Kissling et al. 2018a, 2018b). A precise inventory of which EBVs are relevant to One Health will offer clarity about where the current integration between bio-surveillance and biodiversity monitoring is the strongest.

## Lesson learned: Data clearinghouses support actionable science

Moving from the integration of biodiversity as a relevant concept to One Health into fruitful interaction between researchers, policy bodies, and the data infrastructure supporting this integration can be done by drawing inspiration from success stories in biodiversity monitoring. Few examples in this field are as successful as the Global Biodiversity Information Facility (GBIF; Rodrigues et al. 2022): By curating and publishing primary biodiversity data in the form of species occurrence records, GBIF is a core component of the capacity of biodiversity scientists to detect (Lajeunesse and Fourcade 2023) and attribute (Gonzalez et al. 2023a) the effects of global changes on biodiversity. The factors contributing to the GBIF’s success as a key resource extend beyond the volume of data. First, being used by the entire biodiversity community, the GBIF serves as a focal point for developing best practices in areas such as data cleaning (Panter et al. 2020), data reconciliation (Spear et al. 2023), as well as the recognition that using the GBIF is now a core professional skill that belongs in the postgraduate curriculum (Parker-Allie et al. 2021). Second, the GBIF is supported by a robust data representation framework that standardizes description of biological entities (Wieczorek et al. 2012) and overarching concepts such as monitoring, niche modeling, and biodiversity assessment (Guralnick et al. 2018). Finally, the use of GBIF data throughout the monitoring-to-action pipeline is supported by their translation into indicators (Jetz et al. 2022) that rely on the notion of EBVs (Pereira et al. 2013) and ecosystem functioning indicators (Pettorelli et al. 2018, Hu et al. 2022), making the GBIF a central point of information to track changes in, and actions on, biodiversity across scales. As it stands, One Health seems to be lacking equivalent mechanisms, and advances in the field of biodiversity monitoring can provide a very efficient template. Nevertheless, it is feasible to build pathogen databases that are interoperable with the GBIF (Stevens et al. 2025), which will accelerate the integration between biodiversity and pathogen monitoring.

## Lesson learned: Monitoring for action happens across scales

The last conceptual ingredient of successful monitoring are bio-diversity observation networks (BONs)—the ensemble of field stations, data and reporting protocols, and governance practices that link *in situ* biodiversity measurements to action and policy recommendations. BONs have been instrumental in tracking the effects of climate change (Wetzel et al. 2015) and provided the early evidence that we were falling behind the Aichi targets. BONs can be tailored to specific ecosystems (Duffy et al. 2013) or regions (Fernández et al. 2015, Takeuchi et al. 2021), thereby ensuring that what is measured is relevant to the scientific practices and the local governance and policy landscape. The federation of BONs into a global biodiversity monitoring system (Gonzalez et al. 2023b) is ongoing work that amplifies the capacity of all stakeholders to track and act on biodiversity change. A multiscale network for biodiversity monitoring with built-in communication and reporting between nodes would be immediately relevant to the surveillance of for example, spillover events at the human–wildlife interface. This integration may be facilitated by the fact that BONs share a natural analogue in One Health networks (Khan et al. 2018, Mwatondo et al. 2023); integration of both concepts, in terms of location, data sharing, and joint reporting, would contribute to rapidly bridging the gap between biodiversity monitoring and biosurveillance. Meanwhile, BONs can also start paying close attention to species that have relevance for health (e.g., hosts of zoonotic pathogens or vector organisms).

## Full circle: From biodiversity monitoring to biosurveillance

It is a very short conceptual step from biodiversity monitoring to biosurveillance. The two fields share practical challenges. First, biodiversity monitoring and biosurveillance both struggle to detect changes at a temporal scale fine enough to guide action, especially when rare events must be detected soon enough to guide concrete actions (Glennon et al. 2019). Second, both fields need to reconcile information at a large enough scale that it can be aggregated into robust indicators to both guide policymaking and track policy efficacy. Finally, biodiversity monitoring and biosurveillance both need to harmonize data sources to streamline the process of deriving insights from these data. Better biosurveillance has benefits in terms of improved human, animal, and plant health, but it also represents a springboard to conduct additional biodiversity monitoring.

As we mentioned above, there is a clear potential to use best practices from biodiversity monitoring to carry out systematic sampling of wildlife hosts and arthropod vectors over space and time, in a way that benefits both fields. In a recent article, an examination of how climate change decreases the relevance of range data for Chagas disease vectors led to clear recommendations about ways to handle long-term data in species distribution modeling (Shirey and Rabinovich 2024). Incorporating more information about reservoirs—about their spatial occupancy (Cobos et al. 2024), spatial dispersal process (Oh et al. 2023), or viral interaction data (Carlson et al. 2022)—has led to more precise and actionable recommendations for sampling. Especially as existing data sets carry within them historical gaps (Cohen et al. 2023), model- and data-driven sampling that mobilizes the best knowledge from biodiversity monitoring can lead to immediate benefits for biosurveillance. This work can also be done at all scales that support the establishment of BONs and, in particular, is compatible with locally optimal networks (Kissling et al. 2024).

Ongoing work at the GBIF should lead to more awareness of the platform and its principles in the health community (Shimabukuro et al. 2024). In 2020, a task group of experts was established to help the GBIF network improve the discovery, access, and the use of biodiversity data of species linked to human diseases (Astorga et al. 2023). Their analysis suggests that the GBIF currently contributes more to broad scale ecological analyses and less to health-related studies. Raising awareness about the usefulness of the platform to the health community would help establish a dialogue around data mobilization. There is no reason to limit these initiatives to human health: Joint disease–biodiversity monitoring can be used to track animal or plant pathogens, in full agreement with the spirit of One Health. Similarly, the Group on Earth Observations BON (GEO BON) has recently initiated an effort to assess the relevance of EBVs to One Health goals (GEO BON2024).

Recent results from biodiversity monitoring support the idea that well-designed monitoring networks support multiple policy objectives (Keatts et al. 2023, Kissling et al. 2024). The One Health quadripartite joint plan of action (The Quadripartite Organizations 2022) called for urgent action on the mainstreaming of environmental expertise. Although this seems like a tall order, we can make immediate progress by reusing the capacity that has been built for the purpose of biodiversity monitoring.

## References

[R1] Astorga F, Groom Q, Shimabukuro PHF, Manguin S, Noesgaard D, Orrell T, Sinka M, Hirsch T, Schigel D (2023). Biodiversity data supports research on human infectious diseases: Global trends, challenges, and opportunities. One Health.

[R2] Carlson CJ (2022). The Global Virome in One Network (VIRION): An atlas of vertebrate-virus associations. MBio.

[R3] Cobos ME, Dunnum JL, Armién B, González P, Juárez E, Salazar JR, Cook JA, Colella JP (2024). Selecting sites for strategic surveillance of zoonotic pathogens: A case study in Panamá. BioRxiv.

[R4] Cohen LE, Fagre AC, Chen B, Carlson CJ, Becker DJ (2023). Coronavirus sampling and surveillance in bats from 1996-2019: A systematic review and meta-analysis. Nature Microbiology.

[R5] Desvars-Larrive A, Vogl AE, Puspitarani GA, Yang L, Joachim A, Käsbohrer A (2024). A One Health framework for exploring zoonotic interactions demonstrated through a case study. Nature Communications.

[R6] Duffy JE, Amaral-Zettler LA, Fautin DG, Paulay G, Rynearson TA, Sosik HM, Stachowicz JJ (2013). Envisioning a marine biodiversity observation network. BioScience.

[R7] Fernández M (2015). Challenges and opportunities for the Bolivian Biodiversity Observation Network. Biodiversity.

[R8] Frederick C, Girard C, Wong G, Lemire M, Langwieder A, Martin M-C, Legagneux P (2021). Communicating with Northerners on the absence of SARS-CoV-2 in migratory snow geese. Écoscience.

[R9] [GEO BON] Group on Earth Observations Biodiversity Observation Network (2024). GEO BON welcomes the new One Health Working Group and Knowledge to Action Hub. GEO BON.

[R10] Glennon EE, Jephcott FL, Restif O, Wood JLN (2019). Estimating undetected Ebola spillovers. PLOS Neglected Tropical Diseases.

[R11] Gonzalez A, Chase JM, O’Connor MI (2023a). A framework for the detection and attribution of biodiversity change. Philosophical transactions of the Royal Society of London. Philosophical Transactions of the Royal Society B.

[R12] Gonzalez A (2023b). A global biodiversity observing system to unite monitoring and guide action. Nature Ecology and Evolution.

[R13] Gonzalez A, Londoño MC (2022). Monitor biodiversity for action. Science.

[R14] Griffith J (2024). BON in a Box: An Open and Collaborative Platform for Biodiversity Monitoring, Indicator Calculation, and Reporting. https://ecoevorxiv.org/repository/view/7941.

[R15] Guralnick R, Walls R, Jetz W (2018). Humboldt core: toward a standardized capture of biological inventories for biodiversity monitoring, modeling and assessment. Ecography.

[R16] Hardisty AR (2019). The Bari manifesto: An interoperability framework for essential biodiversity variables. Ecological Informatics.

[R17] Hu Z, Dakos V, Rietkerk M (2022). Using functional indicators to detect state changes in terrestrial ecosystems. Trends in Ecology and Evolution.

[R18] Jetz W, McGowan J, Rinnan DS, Possingham HP, Visconti P, O’Donnell B, Londoño-Murcia MC (2022). Include biodiversity representation indicators in area-based conservation targets. Nature Ecology and Evolution.

[R19] Keatts L, Ondzie A, Perrin M, Cournarie M, Olson S (2023). A wildlife mortality monitoring network that promotes human and wildlife health. One Health Cases.

[R20] Khan MS, Rothman-Ostrow P, Spencer J, Hasan N, Sabirovic M, Rahman-Shepherd A, Shaikh N, Heymann DL, Dar O (2018). The growth and strategic functioning of One Health networks: A systematic analysis. Lancet Planetary Health.

[R21] Kissling DW (2024). Towards a modern and efficient European biodiversity observation network fit for multiple policies. https://ecoevorxiv.org/repository/view/7993.

[R22] Kissling WD (2018a). Building essential biodiversity variables (EBVs) of species distribution and abundance at a global scale: Building global EBVs. Biological Reviews.

[R23] Kissling WD (2018b). Towards global data products of Essential Biodiversity Variables on species traits. Nature Ecology and Evolution.

[R24] Lajeunesse A, Fourcade Y (2023). Temporal analysis of GBIF data reveals the restructuring of communities following climate change. Journal of Animal Ecology.

[R25] Leadley P (2022). Achieving global biodiversity goals by 2050 requires urgent and integrated actions. One Earth.

[R26] Mace GM, Barrett M, Burgess ND, Cornell SE, Freeman R, Grooten M, Purvis A (2018). Aiming higher to bend the curve of biodiversity loss. Nature Sustainability.

[R27] Maney C, Guaras D, Harrison J, Guizar-Coutiño A, Harfoot MBJ, Hill SLL, Burgess ND, Sutherland W (2024). National commitments to Aichi Targets and their implications for monitoring the Kunming-Montreal Global Biodiversity Framework. NPJ Biodiversity.

[R28] Mwatondo A (2023). A global analysis of One Health networks and the proliferation of One Health collaborations. Lancet.

[R29] Nature (2020). The United Nations must get its new biodiversity targets right. Nature.

[R30] Oh G, Aravamuthan S, Ma TF, Mandujano Reyes JF, Ballmann A, Hefley T, McGahan I, Russell R, Walsh DP, Zhu J (2023). Model-based surveillance system design under practical constraints with application to white-nose syndrome. Environmental and Ecological Statistics.

[R31] Panter CT, Clegg RL, Moat J, Bachman SP, Klitgård BB, White RL (2020). To clean or not to clean: Cleaning open-source data improves extinction risk assessments for threatened plant species. Conservation Science and Practice.

[R32] Parker-Allie F (2021). Towards a post-graduate level curriculum for biodiversity informatics: Perspectives from the Global Biodiversity Information Facility (GBIF) community. Biodiversity Data Journal.

[R33] Pereira HM (2013). Essential biodiversity variables. Science.

[R34] Pettorelli N (2018). Satellite remote sensing of ecosystem functions: Opportunities, challenges and way forward. Remote Sensing in Ecology and Conservation.

[R35] Rodrigues A (2022). Primary biodiversity data and the Post-2020 Global Biodiversity Framework. Global Biodiversity Framework.

[R36] Royaux C (2024). Guidance framework to apply good practices in ecological data analysis: Lessons learned from building Galaxy-Ecology. Gigascience.

[R37] Schmeller DS (2017). An operational definition of essential biodiversity variables. Biodiversity and Conservation.

[R38] Schwantes AM, Firkowski CR, Affinito F, Rodriguez PS, Fortin M-J, Gonzalez A (2024). Monitoring ecosystem services with essential ecosystem service variables. Frontiers in Ecology and the Environment.

[R39] Shimabukuro P, Groom Q, Fouque F, Campbell L, Chareonviriyaphap T, Etang J, Manguin S, Sinka M, Schigel D, Ingenloff K (2024). Bridging biodiversity and health: The Global Biodiversity Information Facility’s initiative on open data on vectors of human diseases. GigaByte.

[R40] Shirey V, Rabinovich J (2024). Climate change-induced degradation of expert range maps drawn for kissing bugs (Hemiptera: Reduviidae) and long-standing current and future sampling gaps across the Americas. Memórias do Instituto Oswaldo Cruz.

[R41] Spear D, van Wilgen NJ, Rebelo AG, Botha JM (2023). Collating biodiversity occurrence data for conservation. Frontiers in Ecology and Evolution.

[R42] Stevens T (2025). A minimum data standard for wildlife disease studies. EcoEvoRxiv.

[R43] Takeuchi Y (2021). The Asia-Pacific Biodiversity Observation Network: 10-year achievements and new strategies to 2030. Ecological Research.

[R44] Tittensor DP (2014). A mid-term analysis of progress toward international biodiversity targets. Science.

[R45] Turak E (2017). Essential biodiversity variables for measuring change in global freshwater biodiversity. Biological Conservation.

[R46] [The Quadripartite Organizations] Food and Agriculture Organization of the United Nations, United Nations Environment Programme, World Health Organization, and World Organisation for Animal Health (2022). World Health Organization, Food and Agriculture Organization of the United Nations.

[R47] Wetzel FT (2015). The roles and contributions of biodiversity observation networks (BONs) in better tracking progress to 2020 biodiversity targets: A European case study. Biodiversity.

[R48] Wieczorek J, Bloom D, Guralnick R, Blum S, Döring M, Giovanni R, Robertson T, Vieglais D (2012). Darwin Core: An evolving community-developed biodiversity data standard. PLOS ONE.

